# ILC2s activated by IL-25 promote antigen-specific Th2 and Th9 functions that contribute to the control of *Trichinella spiralis* infection

**DOI:** 10.1371/journal.pone.0184684

**Published:** 2017-09-12

**Authors:** Pornpimon Angkasekwinai, Wichuda Sodthawon, Siranart Jeerawattanawart, Adithap Hansakon, Kovit Pattanapanyasat, Yui-Hsi Wang

**Affiliations:** 1 Department of Medical Technology, Faculty of Allied Health Sciences, Thammasat University, Pathumthani Thailand; 2 Graduate Program in Biomedical Sciences, Faculty of Allied Health Sciences, Thammasat University, Pathumthani Thailand; 3 Center of Excellence for Flow Cytometry, Office for Research and Development, Faculty of Medicine, Siriraj Hospital, Mahidol University, Bangkok, Thailand; 4 Division of Allergy and Immunology, University of Cincinnati, Cincinnati Children’s Hospital Medical Center, Cincinnati, OH United States of America; McGill University, CANADA

## Abstract

IL-25, an IL-17 family cytokine, derived from epithelial cells was shown to regulate Th2- and Th9-type immune responses. We previously reported that IL-25 was important in promoting efficient protective immunity against *T*. *spiralis* infection; however, the cellular targets of IL-25 to elicit type-2 immunity during infection have not yet been addressed. Here, we investigated IL-25-responding cells and their involvement in mediating type-2 immune response during *T*. *spiralis* infection. ILC2 and CD4^+^ Th2 cells residing in the gastrointestinal tract of *T*. *spiralis* infected mice were found to express high levels of surface interleukin-17 receptor B (IL-17RB), a component of the IL-25 receptor. Following *T*. *spiralis* infection, activated ILC2s upregulated surface MHCII expression and enhanced capacity of effector T helper cell in producing antigen-specific Th2 and Th9 cytokines through MHCII-dependent interactions. Reciprocally, lack of CD4^+^ T helper cells impaired ILC2 function to produce type 2-associated cytokines in responding to IL-25 during *T*. *spiralis* infection. Furthermore, mice deficient in IL-17RB showed markedly reduced ILC2 numbers and antigen-specific Th2 and Th9 cytokine production during *T*. *spiralis* infection. The *Il17rb*^-/-^ mice failed to mount effective antigen specific Th2 and Th9 functions resulting in diminished goblet cell and mast cell responses, leading to delayed worm expulsion in the intestines and muscles. Thus, our data indicated that ILC2s and CD4^+^ Th2 cells are the predominant cellular targets of IL-25 following *T*. *spiralis* infection and their collaborative interactions may play a key role in mounting effective antigen-specific Th2 and Th9 cytokine responses against *T*. *spiralis* infection.

## Introduction

Different tissue-dwelling parasitic nematodes that possess distinct intestinal niches and life cycles may confer different susceptibility to specific immune cell effector functions. *Trichinella spiralis* is a parasitic nematode that inhabits both the small intestinal and striated muscle cells after being consumed in larva-contaminated meat [1]. Several effector innate immune cells such as mast cells, eosinophils and goblet cells appear to participate in eliciting the immune response against *T*. *spiralis* infection [2]. While the mechanisms to mount the immune response against *T*. *spiralis* infection involve both T cell-dependent and -independent pathways [3], effective expulsion of the *T*. *spiralis* worm requires CD4^+^ T cells [4].

CD4^+^ Th2 cells, producing IL-4, IL-5 and IL-13 cytokines, have been recognized as the key immune cell type to control gastrointestinal helminth infection. Parallel to the adaptive CD4^+^ Th2 cells, recently identified type-2 innate lymphoid cells (ILC2s) have been shown to be the primary producers of the signature cytokines IL-5 and IL-13 in the early stage of the immune response [5–7]. IL-13, derived from ILC2s, was shown to regulate tuft and goblet cell expansion [8], while IL-5 secreted from ILC2 cells plays a role in promoting eotaxin production and eosinophil accumulation [9]. ILC2s lack antigen specific receptors and express receptors for cytokines, including IL-2, IL-7, TSLP, IL-33, and IL-25 [10]. Several studies have demonstrated that ILC2s induce an immune response against nematode *N*. *brasiliensis* helminth infection [10–12]; whether ILC2s play a key role in expelling *T*. *spiralis* worm infection remains unclear.

IL-25, a member of the IL-17 family, is known to be involved in mediating type-2 immunity against gastrointestinal helminth infection. The induction of IL-25 was observed in the intestines of mice infected with several helminthes, including *N*. *brasiliensis*, *T*. *muris* and *H*. *polygyrus bakeri* [2]. After *N*. *brasiliensis* infection, intestinal IL-25 production was upregulated, possibly by tuft cells [8, 13], which activated resident ILC2s, resulting in worm expulsion, even in the absence of an adaptive immune response. Thus, mice deficient in IL-17RB, a cognate receptor for IL-25, failed to mount an effective immune response to *N*. *brasiliensis* due to delayed and inefficient type-2 cytokine production [5, 14, 15]. While induction of intestinal IL-25 was also shown to be upregulated during *Trichuris muris* infection, IL-25-mediated immune response to this helminth appeared to be lymphocyte dependent [16].

Epithelial cell-derived IL-25 has also been demonstrated to be a key player in eliciting type-2 immune response to *T*. *spiralis* infection [17]. However, the cellular target responsible for IL-25 function and the involvement of ILC2s in response to *T*. *spiralis* infection have not been investigated. In this study, we showed that ILC2s and CD4^+^ Th2 cells were the primary immune cells that expressed surface IL-17RB during *T*. *spiralis* infection. Co-cultured ILC2s with effector/memory T cells augmented the production of antigen-specific Th2 and Th9 cytokines. Depletion of adaptive CD4^+^ T cells resulted in impaired ILC2 function in response to IL-25 stimulation resulting in reduced production of type 2-associated cytokines and IL-9. Furthermore, mice deficient in IL-17RB exhibited reduced ILC2 number and impaired Th2 and Th9 functions, resulting in delayed *T*. *spiralis* clearance. Our studies suggest that reciprocal effect between CD4^+^ and ILC2 cells in response to IL-25 mounts an effective Th2 and Th9 cell-specific immune response against *T*. *spiralis* infection.

## Materials and methods

### Animals

BALB/c mice were obtained from The National Laboratory Animal Center, Mahidol University. Female 6- to 8-week-old mice were used for the experiments. All genetically modified mice, including; IL-4-GFP reporter (4GET), *Il17rb*^-/-^, and *Il17rb*^-/-^/4GET strains, used in this study were backcrossed to the BALB/c background for more than 10 generations [18]. All mice were bred and housed under specific pathogen-free conditions in the animal facility of the Thammasat University. At the end of the experiment, the mice were euthanized by controlled gradual displacement with carbon dioxide using a flow meter in accordance with IACUC and the American Veterinary Medicine Association (AMVA) guidelines on euthanasia. All animal studies were approved by the Thammasat University Animal Care and Use Committee.

### Monoclonal antibodies

PE-conjugated anti-CD127 (A7R34), FITC-conjugated ani-KLRG1 (2F1/KLRG1), V500-conjugated anti-CD4 (GK1.5), allophycocyanin (APC)-Cy7-conjugated anti-Thy1.2 (30-H12), PE-Cy7-conjugated anti-CD3e (145-2C11), PE-conjugated anti-CD62L (MEL-14), APC-conjugated anti-CD44 (IM7), biotinylated anti-T1/ST2 (RMST2-33), APC-Cy7-conjugated anti-c-KIT (2B8), PE-conjugated anti-I-A/I-E (major histocompatibility complex class II [MHC-II]) (clone M5/114.15.2), PerCP-Cy5.5-conjugated mAbs against lineage markers (CD11b [M1/70], CD11c [HL3], Gr.1 [RB6-8C5], CD49b [DX5], CD8 [53–6.7], B220 [RA3-6B2], CD335 [NKP46, 29A.4]) antibodies were purchased from e-Bioscience. Brilliant Violet 421-labeled Streptavidin was purchased from BioLegend, and anti-IL-17RB antibody (clone SS13B) obtained from Dr. Yui-Hsi Wang, University of Cincinnati, Cincinnati Children’s Hospital Medical Center) was conjugated with APC using Zenon Alexa Fluor 647 mouse IgG1 labelling Kit (Thermo Fisher Scientific).

### Lamina propria (LP) cell isolation

The small intestines were harvested, and mesentery, adipose tissue, and peyer’s patches were removed. The small intestines were then opened longitudinally, cut into small pieces and washed with PBS, followed by vigorously shaking in HBSS with 10% FCS, 20 μM Hepes, 5 mM EDTA, 100 U/ml penicillin, 100 μg/ml streptomycin, 1 mM DTT, for 30 min at 37°C to remove epithelial and intraepithelial cells. The tissues were then washed with HBSS and digested with 1 mg/ml collagenase A (Roche, Mannheim, Germany), 0.05 mg/ml liberase, and 0.125 mg/ml DNase I (Roche) at 37°C for 30 minutes. Digested cells were then subjected to Percoll centrifugation (37.5%), and LP leukocytes were isolated from the interface, and after washing, resuspended in medium for subsequent analysis.

### Flow cytometric analysis and cell sorting

To analyze ILC2s and Th2 cells, LP or mesenteric lymph node cells were stained with fluorochrome-conjugated antibodies against CD3e, CD4, CD127, Thy1.2, c-KIT, KLRG1, IL-17RB, T1/ST2, and a combination of lineage (Lin) markers, including CD11b, CD11c, B220, CD49b, Gr-1, CD335, and CD8. Stained cells were fixed, and analyzed using the BD FACSVerse™ cytometer (BD Biosciences), and the acquired data were analyzed with the FlowJo Software (Treestar) as described [18]. For cell sorting experiments, mesenteric lymph node cells were enriched after the removal of lineage positive cells labeled with microbeads conjugated with mAbs against CD11b, CD8, and CD19 using a MACS column (Miltenyi Biotech, Germany). Enriched Lin^-^ cells were stained with lineage markers (CD11b, CD11c, Gr.1, B220, CD335), CD3e, CD4, IL-17RB, T1/ST2 and CD127 antibodies. Lin^-^CD3^-^CD4^-^IL-17RB^+^CD127^+^T1/ST2^+^ cells were sorted for ILC2. For effector/memory CD4^+^ T cell purification, CD3^+^CD4^+^CD62L^-^CD44^+^ were sorted using a FACSAria II (BD Biosciences). For further characterization of surface MHCII expression, Lin^-^CD4^-^CD4^-^IL-17RB^+^CD127^+^T1/ST2^+^GFP^+^ cells were gated and assessed for MHCII expression by flow cytometry.

### Parasite infection

*T*. *spiralis* (ISS62) originated from an outbreak in Mae Hong Son Province in 1986 [19] and was maintained in ICR mice. The larvae were recovered from muscles of infected mice after 30-day post infection by pepsin-acid digestion. Mice were orally gavaged with 400 *T*. *spiralis* larvae and euthanized at various time points after infection.

### CD4+ T cell depletion

For CD4^+^ T cell depletion, mice were intraperitoneally injected with anti-CD4 mAb (clone GK1.5, 300 μg/mouse) or control rat IgG (300 μg/ mouse) every other day after infection. Mesenteric lymph node or lamina propria cells isolated from the small intestine of antibody-treated mice infected for 7 and 14 days were used to analyze ILC2 cell frequency using flow cytometry. Mesenteric lymph node cells of antibody-treated mice infected for 7 days were cultured in the presence or absence of IL-25 (10 ng/ml, R&D Systems) with or without *T*. *spiralis* antigen (10 μg/ml). To measure cytokines from ILC2s, ILC2s were enriched after depleting lineage positive cells labeled with microbeads conjugated with mAbs against CD11b, CD8, CD4, B220 and CD19 using a MACS column (Miltenyi Biotech, Germany) [18]. Enriched cells were stimulated with IL-25 (10 ng/ml, R&D Systems) or phorbol myristate acetate (PMA) (50 ng/ml, Sigma) plus ionomycin (500 ng/ml, Sigma). After stimulating 72 hours, supernatants were collected and the amounts of secreted cytokines were measured by ELISA.

### Worm burden analysis

To analyze intestinal worm burden, the small intestines were collected, opened longitudinally and incubated in Hanks’ Balanced Salt Solution (HBSS) at 37°C for 3 hr [20]. Following incubation, the intestines were agitated; the worms were then counted using an inverted microscope. Muscle larvae burden was assessed by digesting whole carcasses of infected mice with pepsin-HCl on day 30 postinfection.

### Histology

Intestinal tissue samples were fixed in 10% buffered formalin, dehydrated in ethanol, and embedded in paraffin wax. The 5-μm sections were stained with periodic acid-Schiff (PAS) to visualize goblet cells [21]. The number of goblet cells was expressed as the amount per 10 villus crypt units (VCU).

### Coculture of effector CD4^+^ T cells and ILC2

To generate ILC2s in this experiment, mice were injected with IL-25Ig (5 μg/mouse) at days 0, 1, 3 and 5 after infection [17, 22]. ILC2s (CD4^-^IL-17RB^+^CD127^+^T1/ST2^+^GFP^+^) were sorted from pooled mesenteric lymph nodes using a cell sorter (FACSAria II, BD Biosciences). To study effector CD4+ T cells, mice were infected and effector CD4^+^ T cells (CD44^+^CD62L^-^CD4^+^CD3^+^ cells) were sorted from the mesenteric lymph nodes from mice infected with *T*. *spiralis* on day 7 postinfection. Sorted ILC2s (5 x 10^4^ cells) were pulsed with 10 μg/ml *T*. *spiralis* extract or OVA antigen for 3 hours at 37°C. Subsequently ILC2s were washed and were then co-cultured with effector CD4^+^ T cells (1 x 10^5^ cells) for 3 days. The antibodies M5/114.15.2 (anti-MHCII, eBioscience) were added to cultures at a concentration of 1 μg/ml. The culture supernatants were collected after 3 days and were then analyzed for cytokine production using ELISA.

### Measurement of cytokine production

Single-cell suspensions of mesenteric lymph nodes harvested from mice infected with *T*. *spiralis* at various time points after infection were enriched before subjection to analysis of antigen-specific cytokine production using ELISA [17]. Briefly, cells were stimulated with *T*. *spiralis* extract antigen at a concentration of 0 or 10 μg/ml. To investigate the effect of IL-25 on cytokine production, cultured cells were treated with IL-25 (10 ng/ml, R&D Systems) in the presence or absence of *T*. *spiralis* antigen stimulation. Supernatants of cultured cells were collected to analyze cytokine production after 72-hour incubation at 37°C with 5% CO2. IL-4, IL-5, IL-9, IFN-γ and IL-17 were analyzed using the antibody pairs from BD Pharmingen, while IL-13 was assessed using ELISA kits (R&D Systems) according to the manufacturer's instructions.

### Real-time RT-PCR analysis

RNA was extracted using TRIzol reagent to serve as templates for cDNA synthesis using oligo-dT, random hexamers and MMLV reverse transcriptase (Invitrogen) [17]. To quantify cytokine gene expression, cDNA samples were amplified in IQ^TM^ SYRB® Green Supermix (Biorad Laboratories) using the primer pairs for targeted cytokines as previously described [17]. Expression levels of target genes were normalized to endogenous actin (*Actb*) transcript levels, and relative quantification of samples was compared with the lowest expression levels serving as the baseline.

### Statistical analysis

Data are presented as mean value ± SD. Data were analyzed using one-way ANOVA with Tukey’s post hoc analysis. Statistical analysis was performed with GraphPad Prism 5 Software. A value of *p* <0.05 was considered significant.

## Results

### Innate lymphoid cell type 2 (ILC2) and CD4^+^Th2 cells express IL-25 receptor during *T*. *spiralis* infection

**Fig 1:**
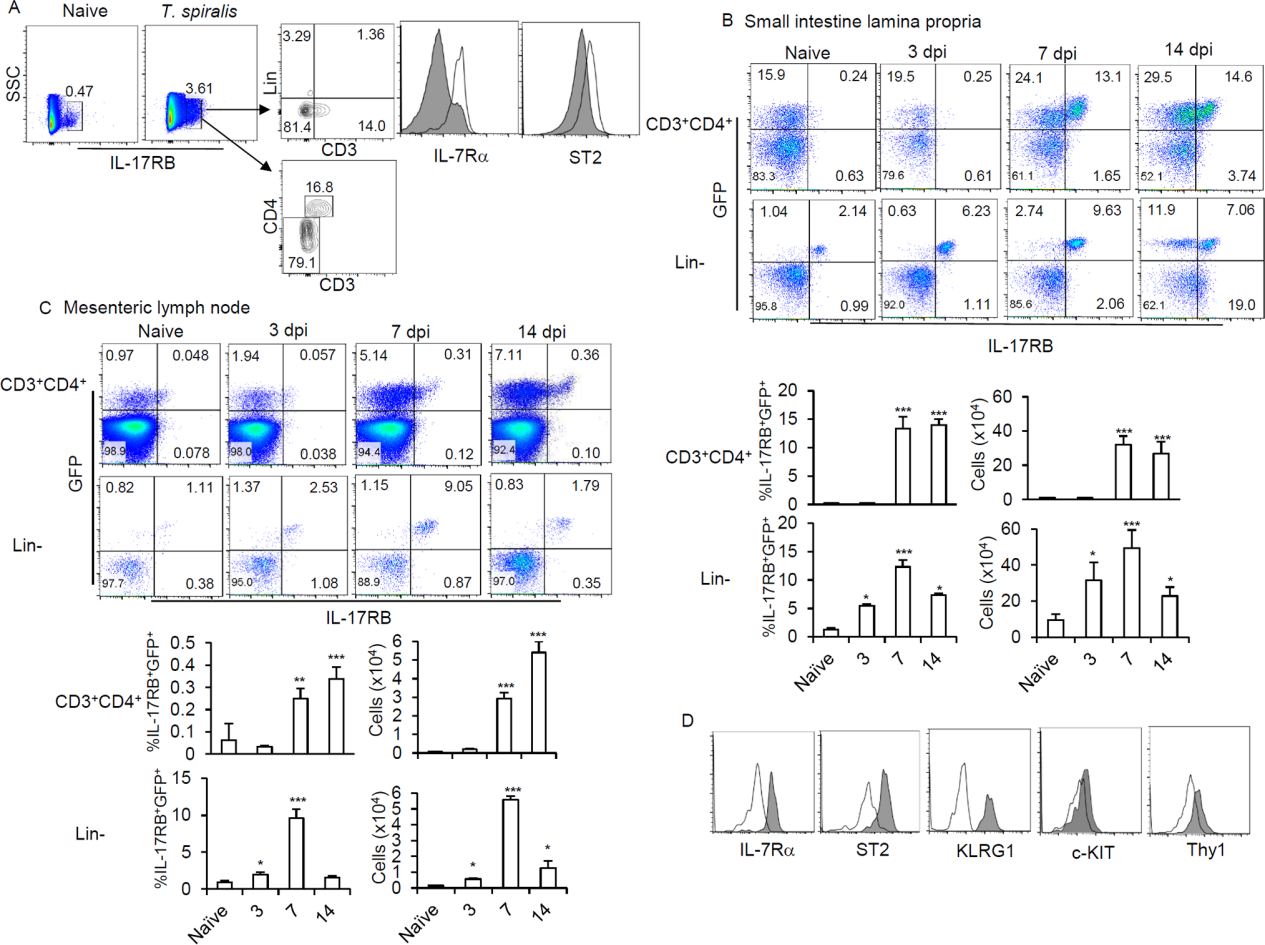
Type 2 innate lymphoid cells (ILC2s) and CD4^+^Th2 cells are the major immune cells expressed IL-25 receptor (IL-17RB) in response to *T*. *spiralis* infection. **(A)** BALB/c mice were infected with *T*. *spiralis* and their lamina propria cells were analyzed for the expression of IL-17RB at day 7 following infection. The gated IL-17RB^+^ population from infected mice was stained with lineage markers (CD11b, CD11c, B220, CD19, DX5, Gr.1, CD8), IL-7Rα, ST2, CD3 and CD4. The expression of IL-7Rα and ST2 on Lin^-^ cells from IL-17RB^+^ (open histogram) were compared with those from IL-17RB^-^ (shaded histogram) population. **(B and C)** Phenotypic analysis and frequencies of IL-17RB^+^GFP^+^ by CD3^+^CD4^+^ and Lin^-^ cells in the lamina propria of the small intestines **(B)** and mesenteric lymph nodes **(C)** of 4GET mice following *T*. *spiralis* infection at days 3, 7 and 14. **(D)** Cell surface marker expression (IL-7 receptor α, KLRG1, ST2, c-KIT and Thy1) in the IL-17RB^+^GFP^+^Lin^-^ cells in the lamina propria of the small intestines after *T*. *spiralis* infection for 7 days. Flow cytometric data shown are a representative histogram profile of one of three independent experiments. The solid gray line indicates the plot profile of isotype-matched control antibody and shaded histogram represents the profile of the indicated antibody. The data represent one of three independent experiments (n = 4 mice each group). Error bars denoted mean ± SD. Significance was determined using one-way ANOVA * p <0.05, ** p <0.01*** and p <0.001 (compared with data in naïve mice).

Related studies have demonstrated that IL-25 functions to promote protective immune response against *T*. *spiralis* infection by augmenting Th2 and Th9 cytokine production [17]; however, the cellular mechanisms underlying IL-25-mediated immune response during infection remain elusive. To identify the immune cells responsible for IL-25-mediated protective immunity against *T*. *spiralis* infection, we examined lamina propria cells from the intestines of mice infected with *T*. *spiralis* using multicolor flow cytometric analysis. Following *T*. *spiralis* infection for 7 days, a population of IL-17RB^+^ cells was found to be increased (Fig 1A). Two dominant populations of cells expressing IL-17RB were identified: cells lacking expression of lineage markers (CD11b, CD11c, B220, CD19, DX5, Gr.1, CD8) and a population of T helper cells (CD3^+^CD4^+^). A distinctive lineage negative cell population (Lin^-^) comprised CD127^+^ and ST2^+^, a phenotype likely to be ILC2s (Fig 1A). Recent studies employing IL-4 reporter mice (4GET) in tracking ILC2 indicated that Lin^-^GFP^+^IL-17RB^+^ cells appeared identical to the IL-25-responding ILC2s [18, 23]. To better understand the involvement of these cells in IL-25-mediated type-2 immunity against *T*. *spiralis* infection, we examined the IL-17RB-expressing cells in the small intestines and mesenteric lymph nodes of mice infected with *T*. *spiralis* at different time periods in 4GET mice using the previously described strategy. After 7 days post *T*. *spiralis* infection, the frequency of CD3^+^CD4^+^ cells that expressed GFP and IL-17RB, previously reported as CD4^+^Th2 cells [24],was significantly increased in both the intestinal lamina propria and draining lymph nodes, and maintained after 14 days (Fig 1B and 1C). Very few GFP^+^IL-17RB^+^CD3^+^CD4^+^ cells could be detected 3 days postinfection or in healthy mice (Fig 1B and 1C). Intriguingly, IL-17RB-expressing Lin^-^ cells that were found to co-express GFP could be readily detected in the lamina propria of the small intestines from uninfected mice, and the frequency of IL-17RB^+^GFP^+^Lin^-^ cells increased significantly in both the lamina propria and draining lymph nodes at day 7 postinfection, but declined at day 14 postinfection (Fig 1B and 1C). Detailed phenotypic characterizations of IL-17RB^+^GFP^+^Lin^-^ cells demonstrated that these cells also expressed the signature markers of recently described ILC2s, including the IL-7 receptor α chain, KLRG1, T1/ST2, c-KIT, and Thy1.2 (Fig 1D). Among a population of Lin^-^ cells, characteristics of ST2^+^IL-17RB^+^KLRG1^+^ ILC2s, but not ST2^-^IL-17RB^+^KLRG1^hi^ or ST2^+^IL-17RB^lo/-^KLRG1^lo^ (described as iILC2 and nILC2 in the lungs, respectively) or ST2^-^IL-17RB^+^IL-7Rα^-^ (MPP^type2^) were induced in the lamina propria of *T*. *spiralis*-infected mice (Fig 1D and S1 Fig). These data suggested that the intestinal ILC2s and CD4^+^Th2 cells are the major cell subsets that express surface IL-17RB, a component of IL-25R, indicating their involvement in IL-25-elicited type-2 immune response during *T*. *spiralis* infection.

### IL-25-activated ILC2s enhance antigen-specific Th2 and Th9 cytokine production

**Fig 2:**
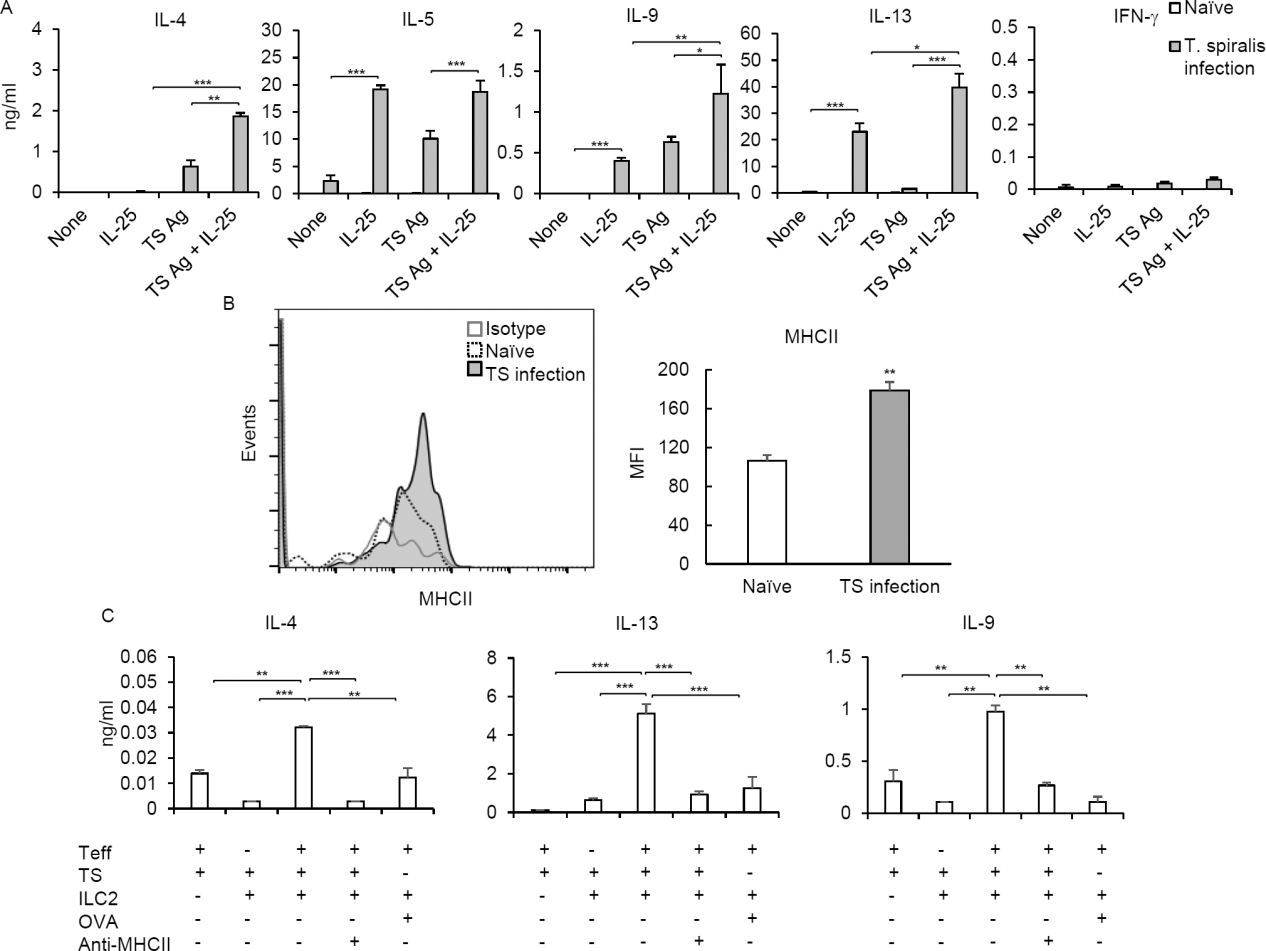
ILC2s induced by IL-25 facilitates antigen-specific Th2 and Th9 cytokine production in response to *T*. *spiralis* infection. **(A)** Mesenteric lymph node cells of naïve mice or mice infected with *T*. *spiralis* stimulated with or without *T*. *spiralis* antigen in the presence or absence of IL-25 for 72 hours were measured for cytokines as indicated and compared. **(B)** Gated ILC2s (Lin^-^CD4^-^IL-17RB^+^CD127^+^T1/ST2^+^GFP^+^) in the lamina propria isolated from the small intestine of naïve and *T*. *spiralis*–infected mice were analyzed for the expression of MHCII and compared. **(C)** ILC2s sorted from IL-25-treated mice and pulsed with *T*. *spiralis* antigen or OVA antigen were co-cultured with or without purified effector/memory CD4^+^ T cells (CD44^+^CD62L^-^CD4^+^CD3^+^) from mesenteric lymph nodes of *T*. *spiralis*–infected mice for 72 hours. An anti-MHCII antibody was added to effector/memory CD4^+^ T cells co-cultured with *T*. *spiralis*-pulsed ILC2 cells. The indicated cytokines in the supernatant were examined by ELISA. Data represented one of three independent experiments (n = 4 mice each group). Error bars denote mean ± SD. Significance was determined by one-way ANOVA * p <0.05, ** p <0.01 and *** p <0.001.

To understand how IL-25 promotes the type-2 immune response during *T*. *spiralis* infection, we first assessed the effect of IL-25 on Th2/Th9 cytokines (IL-4, IL-5, IL-9 and IL-13) and IFN-γ production by mesenteric lymph node cells from 7-day *T*. *spiralis*-infected mice in the presence or absence of *T*. *spiralis* antigen stimulation. Consistent with other studies [24], IL-25 alone is sufficient to induce robust IL-5 and IL-13 and moderate IL-9 but little IL-4 and IFN-γ production; whereas, *T*. *spiralis* antigens triggered primarily IL-4, IL-5 and IL-9, but little IL-13 and IFN-γ production by mesenteric lymph node cells from *T*. *spiralis*-infected mice (Fig 2A). Notably, IL-25 plus *T*. *spiralis* antigens induced large amounts of IL-4, IL-5, IL-9 and IL-13 production by mesenteric lymph node cells of *T*. *spiralis*-infected mice (Fig 2A). Thus, IL-25 could enhance *T*. *spiralis* antigen-specific Th2 cytokine and IL-9 production by CD4^+^ cells that were accumulated together with the IL-17RB-expressing ILC2s in the mesenteric lymph nodes 7 days post *T*. *spiralis* infection (Fig 1C).

Accumulated evidences from related studies suggest that ILC2s may promote antigen-specific Th2 immune responses by enhancing CD4^+^ T cell function through surface MHCII expression [12, 24]. To determine whether ILC2s have the potential to regulate intestinal CD4^+^ Th2 and Th9 cell functions during *T*. *spiralis* infection, we first examined and compared MHC class II expression by ILC2s from *T*. *spiralis* infected mice with those from healthy mice. Indeed, we found that intestinal IL-17RB-expressing ILC2s from mice infected with *T*. *spiralis* expressed higher levels of surface MHC class II, compared with those from uninfected mice, implying that intestinal ILC2s may be activated to acquire the capability to potentiate CD4^+^ T cell function during *T*. *spiralis* infection (Fig 2B).

Next, we tested whether ILC2s may function in promoting antigen-specific T helper cell response by coculturing ILC2s and CD4^+^ T effector/memory cells, isolated from 4-GET mice infected with *T*. *spiralis* for 7 days when both ILC2s and CD4^+^ Th2 cells were induced (Fig 1B). While effector/memory CD4^+^ T cells or ILC2s alone stimulated with *T*. *spiralis* antigen produced low levels of Th2 cytokines, co-cultured ILC2s, pulsed with *T*. *spiralis* antigen (not an unrelated control OVA antigen), induced antigen-specific effector/memory cells to produce significantly large amounts of Th2 cytokine, IL-4 and IL-13, as well as Th9 cytokine, IL-9 (Fig 2C). More importantly, a neutralizing anti-MHCII antibody prevented the enhanced antigen-specific cytokine production of effector/memory co-cultured with *T*. *spiralis*-pulsed ILC2. By contrast, very little antigen-specific IFN-γ production could be detected in the supernatants of *T*. *spiralis* antigen-stimulated CD4^+^ effector/memory T cells, ILC2s or co-cultured ILC2s and CD4^+^ effector/memory T cells. These data suggest that *T*. *spiralis* infection induces the accumulation and activation of intestinal ILC2s that can promote antigen-specific Th2 and Th9 cell functions by enhancing their cytokine production through an MHCII-dependent interaction.

### Absence of CD4^+^ T cells during *T*. *spiralis* infection resulted in an impaired function of ILC2s

**Fig 3:**
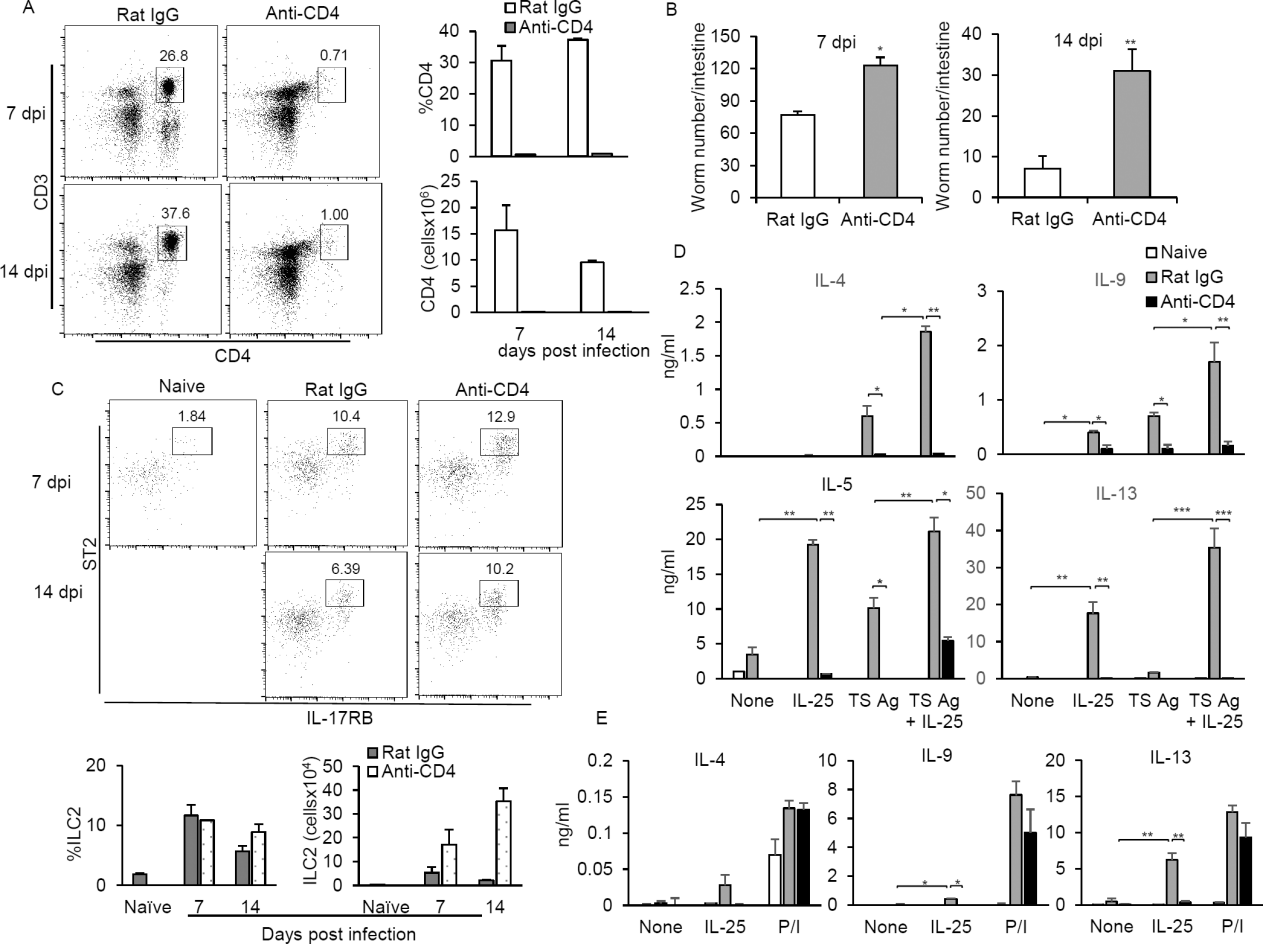
ILC2s failed to function in *T*. *spiralis*–mice ablated CD4+ T cells. BALB/c mice were intraperitoneally given anti-CD4 or rat IgG antibody every other day after infection with *T*. *spiral*is. **(A)** Frequency and numbers of CD3^+^CD4^+^ cells at 7 and 14 days postinfection were evaluated in the mesenteric lymph node of infected mice treated with anti-CD4 or rat IgG antibody. **(B)** At 7 and 14 days postinfection, the small intestines were harvested and subjected for worm burden. **(C)** Detection and frequency of ILC2s (CD3^-^CD4^-^IL-17RB^+^CD127^+^T1/ST2^+^) in the mesenteric lymph nodes of infected mice treated with anti-CD4 or rat IgG antibody. **(D)** Mesenteric lymph node cells of *T*. *spiralis*-infected mice treated with anti-CD4 or rat IgG antibody were treated with IL-25 with or without *T*. *spiralis* antigen for 72 hours and the indicated cytokines in the supernatant were assessed using ELISA. **(E)** Lin^-^ cells from the mesenteric lymph node of *T*. *spiralis*-infected mice treated with anti-CD4 or rat IgG antibody were enriched using a MACS column. The enriched ILC2 cells were then restimulated with IL-25 or PMA/ionomycin for 72 hours, and the indicated cytokines in the supernatant were assessed using ELISA. Data represented one of three independent experiments (n = 4 mice each group). Error bars denote mean ± SD. Significance was determined by one-way ANOVA * p <0.05, ** p <0.01 and *** p <0.001.

Related studies demonstrated that mice lacking CD4^+^ T cells failed to mount an effective protective immune response against *T*. *spiralis* infection [4, 25], suggesting that ILC2s alone may be insufficient to elicit robust Th2 immune response. To investigate whether the presence of CD4^+^ T cells regulate the induction and function of ILC2s, we analyzed the mesenteric lymph node and intestinal ILC2s from mice which were intraperitoneally injected with anti-CD4 antibody every other day after *T*. *spiralis* infection. Indeed, anti-CD4 antibody treatment resulted in effective reduction of CD4^+^ T cell number to less than 1% of total mononuclear cells in the mesenteric lymph nodes and lamina propria of mice after *T*. *spiralis* infection (Fig 3A and S2A Fig). Consequently, the loss of CD4^+^ T cells in *T*. *spiralis*-infected mice treated with anti-CD4, not isotype control antibody, resulted in the failure of effective worm expulsion in the intestines, as evidenced by the sustained worm number at day 14 postinfection (Fig 3B). Compared with infected mice treated with isotype control antibody, the percentage and number of ILC2s remained intact in the mesenteric lymph nodes and the lamina propria of *T*. *spiralis*-infected mice that were treated with anti-CD4 antibody (Fig 3C and S2B Fig). Intriguingly, IL-25-enhanced IL-5, IL-9 and IL-13 production was less and antigen-specific Th2 and Th9 responses in mesenteric lymph nodes of CD4 mAb-treated mice at day 7 postinfection were significantly fewer than those of isotype mAb-treated mice, indicating that despite ILC2s remaining intact after depleting CD4+ T cells, these cells responded poorly to IL-25 stimulation (Fig 3D). To confirm the attenuated ILC2 activation in response to IL-25 in mice ablated of CD4^+^ T cells, ILC2s were enriched by depleting lineage positive cells labeled with microbeads conjugated with mAbs against CD11b, CD8, CD4, B220 and CD19 and then stimulated with IL-25 or with PMA/Ionomycin. While treatment with PMA/Ionomycin in enriched ILC2s from mice treated with control and anti-CD4 antibody induced comparable level of cytokines IL-4, IL-9 and IL-13, stimulation with IL-25 significantly enhanced the secretion of IL-9 and IL-13 in the enriched ILC2s from isotype mAb-treated mice but not CD4 mAb-treated mice (Fig 3E). These data suggest that CD4^+^ T cells are nonessential to induce ILC2s during the immune response to *T*. *spiralis* infection, but are required for ILC2 function to respond to IL-25 stimulation during *T*. *spiralis* infection.

### Lack of IL-25 receptor diminishes ILC2 induction and antigen-specific Th2 and Th9 functions

**Fig 4:**
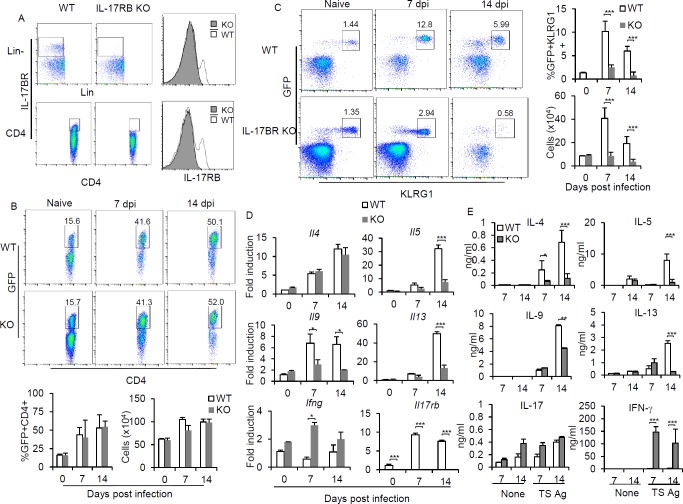
Signaling through IL-17RB is essential to induce ILC2 and antigen specific Th2 and Th9 responses. *Il17rb*^-/-^/4GET or wild type 4GET mice were infected with *T*. *spiral*is. **(A)** Detection of IL-17RB in lineage negative (CD11b^-^CD11c^-^B220^-^CD49b^-^Gr.1^-^CD335^-^CD3^-^CD4^-^CD8^-^) and CD3^+^CD4^+^ T cells. **(B)** Frequency and numbers of CD3^+^CD4^+^GFP^+^ cells at 7 and 14 days postinfection were evaluated in the mesenteric lymph node of infected *Il17rb*^-/-^/4GET or wild type mice. **(C)** Detection and frequency of ILC2s (CD3^-^CD4^-^CD127^+^T1/ST2^+^KLRG1^+^GFP^+^) in the lamina propria of the small intestines of *Il17rb*^-/-^/4GET or wild type 4GET mice infected with *T*. *spiral*is at 7 and 14 days postinfection. **(D)** Effector/memory (CD3^+^CD4^+^CD44^+^) T cells from mesenteric lymph nodes of *T*. *spiralis*-infected wild type and *Il17rb*^*-/-*^ mice were sorted and subjected to analyze the expression of indicated cytokine using real-time PCR. Data are expressed as fold induction over actin (*Actb*) expression, with the mRNA levels in the naïve group set as 1. **(E)** At day 7 and 14 postinfection, mesenteric lymph node cells were harvested and single cell suspensions were then cultured with or without *T*. *spiralis* extract antigen (10 μg/ml). After three days, the supernatant was collected and analyzed for *T*. *spiralis*-specific cytokine production by ELISA. Graphs depict mean±SD and are a representative of at least three independent experiments with three to four mice each group. Significance was determined by one-way ANOVA with Tukey’s post hoc analysis * p <0.05, ** p <0.01 and *** p <0.001.

To examine the requirements of the IL-25 signal in regulating the induction and function of IL-17RB-expressing ILC2s and CD4^+^ Th2 cells during *T*. *spiralis* infection, BALB/c *Il17rb*^-/-^/4GET mice were generated. As expected, IL-17RB-expressing cells could be readily detected in wild type mice after *T*. *spiralis* infection, but not in *Il17rb*^*-/-*^ mice (Fig 4A). While the number of GFP^+^CD4^+^ cells were comparable between wild type 4GET and *Il17rb*^-/-^/4GET mice at 7 or 14 days after *T*. *spiralis* infection (Fig 4B), the loss of IL-17RB signals resulted in a significant reduction in the number of GFP^+^KLRG1^+^ILC2s in mice infected with *T*. *spiralis* (Fig 4C). Intriguingly, the expression levels of *Il5*, *Il9*, and *Il13* transcripts by the sorted GFP^+^CD4^+^ T effector/memory cells that lacked IL-17RB signals were markedly reduced despite a comparable number of GFP^+^CD4^+^ cells (Fig 4D). In contrast, the expression levels of *Ifn*γ transcription by GFP^+^CD4^+^ T effector/memory cells from *Il17rb*^*-/-*^ mice were significantly higher than those by these cells from wild type mice (Fig 4D). Furthermore, mesenteric lymph node cells from *T*. *spiralis*-infected *Il17rb*^*-/-*^ mice produced much lower amounts of antigen-specific IL-5, IL-9 and IL-13 cytokines, but larger amounts of IFN-γ than those from infected wild type mice (Fig 4E). Although the expression levels of *Il4* transcript in GFP^+^CD4^+^ T effector/memory cells were similar between wild type and IL-17RB-deficient mice, antigen-specific IL-4 production by mesenteric lymph node cells was significantly reduced when mice lacking IL-17RB signals during *T*. *spiralis* infection (Fig 4E). Collectively, these data suggest that the IL-25 receptor signal is required for ILC2s accumulation during infection, which in turn may enable the capability of CD4+TH effector cells to elicit strong antigen-specific Th2 and Th9 cytokine responses against *T*. *spiralis* infection. Our studies did not exclude the possibility that attenuated antigen-specific Th2 cytokine production in IL-17RB-deficient mice may result from the intrinsic defect of IL-25-mediated Th2 and Th9 cytokine production as previously described [22, 26, 27].

### IL-25 receptor deficiency attenuates Th2/Th9 immune responses that results in defective *T*. *spiralis* worm clearance

**Fig 5:**
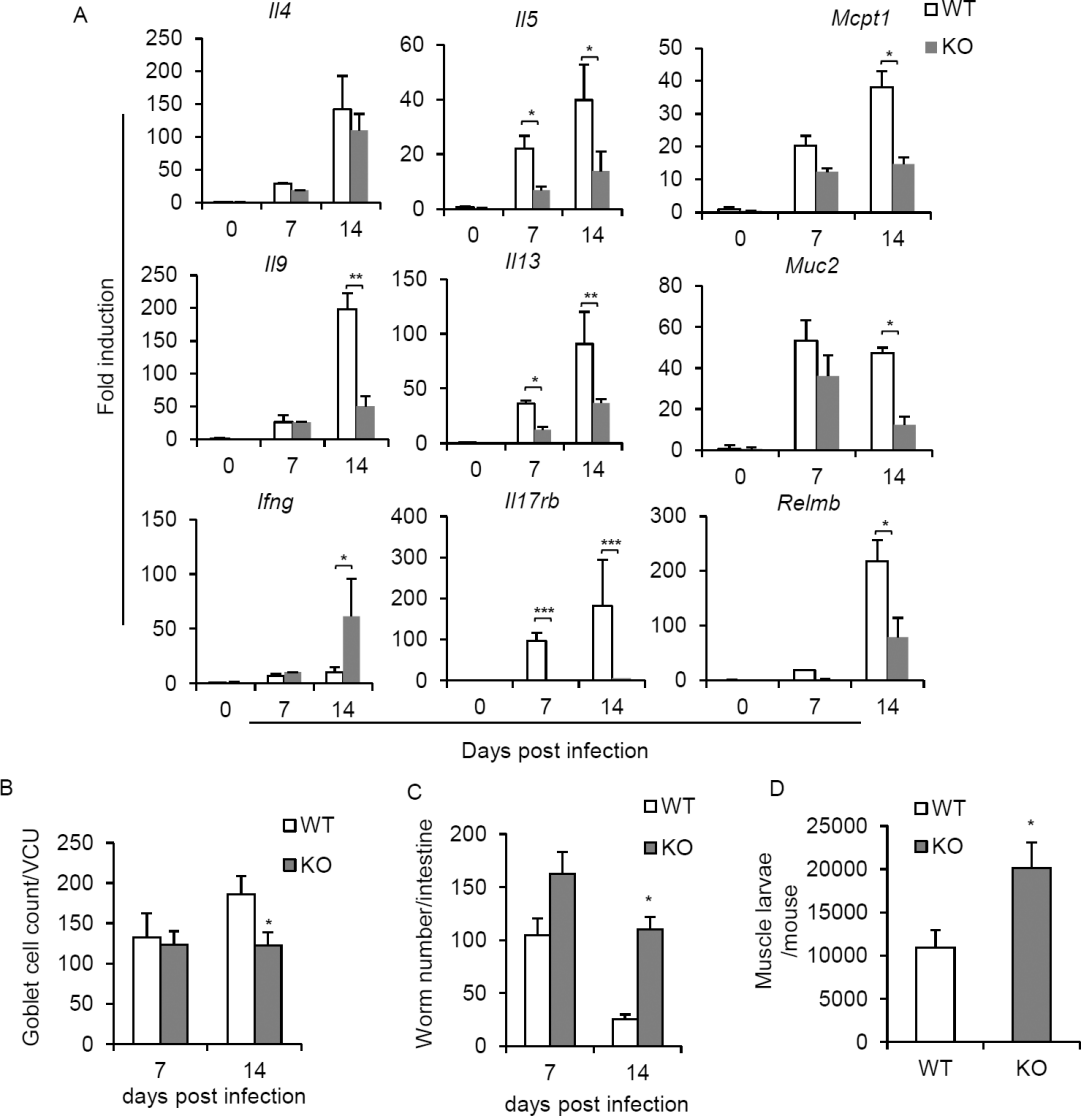
Absence of IL-17RB resulted in reduced intestinal Th2 and Th9 effector responses and attenuated intestinal and muscle *T*. *spiral*is worm clearance. *Il17rb*^-/-^ or BALB/c mice were infected with *T*. *spiral*is. **(A)** At days 7 and 14 postinfection, the small intestines (jejunum) were harvested and subjected to RNA extraction, followed by cDNA synthesis and cytokine gene expression using real-time PCR analysis. Data are expressed as fold induction over actin (*Actb*) expression, with the mRNA levels in the naïve group set as 1. **(B)** The small intestines (jejunum) were fixed with 10% formalin buffer and subjected to histological analysis of goblet cells by Periodic acid–Schiff (PAS) staining. Numbers of goblet cells were expressed per villus crypt unit (VCU). **(C)** At days 7 and 14 postinfection, the whole intestines of *T*. *spiral*is-infected *Il17rb*^-/-^ or BALB/c mice were harvested and analyzed for adult worms. **(D)** At day 30 postinfection, whole carcasses of infected mice from *Il17rb*^-/-^ or wild type groups were analyzed for muscle larvae burden. Graphs depict mean±SD and are a representative of at least three independent experiments with four mice each group. Significance was determined using one-way ANOVA with Tukey’s post hoc analysis * p <0.05, ** p <0.01 and *** p <0.001.

Having demonstrating the role of the IL-25 receptor signal in promoting ILC2s and antigen specific T cell immune response during *T*. *spiralis* infection, we examined its involvements in *T*. *spiralis* worm clearance. After 14 days of *T*. *spiralis* infection, the expression levels of *Il5*, *Il9* and *Il13* transcripts in the small intestine of *Il17rb*^*-/-*^ mice were significantly lower than those of the wild type mice (Fig 5A). Consequently, mice lacking IL-25 signals expressed much lower levels of *Muc2*, *Relmb*, and *Mcpt1* genes, which were shown to be positively regulated by Th2 cytokine and IL-9 effector responses (Fig 5A). Compared with the wild type mice, fewer numbers of goblet cells could be observed in the small intestines of *Il17rb*^*-/-*^ mice at day 14 postinfection (Fig 5B). Thus, lack of the IL-25 receptor signal dampens Th2 and Th9 immune responses, resulting in attenuated functions of goblet cells, epithelial cells and mast cells in the intestines during *T*. *spiralis* infection.

Next, we investigated the effect of IL-25 receptor deficiency in *T*. *spiralis* worm clearance. Although much fewer ILC2s could be detected in *Il17rb*^*-/-*^ mice than those in the wild type mice on day 7 postinfection (Fig 4B), no significant difference was observed in *T*. *spiralis* worm numbers between *Il17rb*^*-/-*^ and wild type mice at this phase of infection (Fig 5C). However, *Il17rb*^*-/-*^ mice were less effective in expelling *T*. *spiralis* than wild type mice, as evidenced by the significant higher adult worm burden in the intestines of *Il17rb*^*-/-*^ mice on day 14 postinfection (Fig 5C), at which the antigen-specific Th2 immune response was markedly diminished (Fig 4B). Furthermore, a higher number of larvae in the muscles could be observed in *Il17rb*^*-/-*^ mice on day 30 postinfection (Fig 5D). Together, these results suggest that the IL-25 receptor signal plays an important role in mounting an effective type-2 immune response contributing to the control of *T*. *spiralis* infection.

## Discussion

Our previous work demonstrated the role of IL-25 in promoting a protective immune response against *T*. *spiralis* infection [17], yet the cellular functional targets in innate and adaptive immunity responsible for the IL-25 function, particularly the involvement of type-2 innate lymphoid cells (ILC2s) in *T*. *spiralis* infection were not clearly addressed. The data presented here showed that the IL-25-mediated protective immune response was primarily driven by the expression of the IL-25 receptor on type-2 innate lymphoid cells (ILC2) and adaptive T helper cells. Further characterization indicated that reciprocal regulation of ILC2 and adaptive T helper cells coordinated to promote IL-25 enhanced antigen-specific Th2 and Th9 cells immune response to *T*. *spiralis* infection. Consequently, IL-25 receptor signaling was required for efficient worm clearance of *T*. *spiralis* infection in both intestinal and muscle phases.

The finding that recently identified type-2 innate lymphoid cells (ILC2s) function as an IL-25 target cell population [5–7] suggests a potential involvement of ILC2s in *T*. *spiralis* infection. Indeed, phenotypic analyses of IL-17RB, a cognate IL-25 receptor expression, showed that ILC2s were the predominant IL-17RB-expressing cells among the innate immune cell types, suggesting that ILC2s were the major innate immune cell types responsible for the IL-25 function in the immune response against *T*. *spiralis* infection. Recent studies indicated the existence of an inflammatory ILC2 (iILC2) population responsive to interleukin 25 (IL-25) and IL-33-responsive natural ILC2 (nILC2) in the lungs of *N*. *brasiliensis*-infected mice [28]. As previously report, the induction of IL-25 responsive cells was transiently induced following *T*. *spiralis* infection. However, unlike IL-25-responsive ILC2s in the lungs of mice infected with *N*. *brasiliensis* that did not express ST2, a majority of ILC2s in the lamina propria of *T*. *spiralis*-infected mice expressed both IL-17RB and ST2, suggesting their abilities in response to IL-25 and IL-33 during the immune response to *T*. *spiralis* infection. Quite possibly, ILC2s may appear in various phenotypes in different anatomical locations. Moreover, the frequencies and function of ILC2s involved in the immune response against different gastrointestinal helminth infection may differ. Additional studies will be required to further define the regulation of tissue ILC2 development and the cooperative function with other IL-25-responsive innate immune cells such as MPP^type2^ in promoting type-2 mediated protective immunity against intestinal helminth infection.

IL-25 has been shown to potentiate the production of type-2 cytokines by Th2 cells [22, 27] and IL-9 by Th9 cells [26] in allergic lung diseases in both mice and humans. Our previous data suggest that IL-25 mediated the protective immunity to *T*. *spiralis* infection by promoting the enhanced antigen-specific Th2 and Th9 cytokine responses [17]. Unlike transient induction of ILC2s during *T*. *spiralis* infection, analyses of IL-17RB expression in adaptive T helper cells revealed that IL-17RB-expressing Th2 cells were induced following 7 days of infection and persisted in the late stage of infection. IL-25 has been demonstrated to be induced on day 1 following *T*. *spiralis* infection [17], which may be followed by promoting the induction of IL-25 responsive ILC2s in the early stage of infection. Related findings suggest that the combined function of MHCII expression by hematopoietic cells and CD4 T cells were required to promote *T*. *spiralis* worm expulsion [4]. Indeed, recent studies have indicated that ILC2 can promote the Th2 immune response in an MHCII-dependent manner [12, 18]. In support of these findings, our results also showed that ILC2 upregulated MHCII expression after stimulation with *T*. *spiralis* infection and these cells pulsed with *T*. *spiralis* antigen could augment antigen-specific type-2 cytokines and IL-9 production by effector/memory T helper cells through an MHCII-dependent mechanism. Therefore, the induction of ILC2s may play an important role in augmenting the function of antigen-specific Th2 and Th9 immune response against *T*. *spiralis* infection. Intriguingly, the function of ILC2s to produce cytokines in response to IL-25 stimulation during *T*. *spiralis* infection required the presence of CD4+ T helper cells since depletion of CD4+ T cells failed to promote IL-25-stimulated ILC2 to produce IL-4, IL-5, IL-9, and IL-13. Our finding suggested that the induced ILC2 and adaptive Th2 and Th9 cells may cooperate to mediate the effective immune response against *T*. *spiralis* infection. Although the mechanism underlying the cooperation of ILC2s and adaptive Th2 and Th9 cells in *T*. *spiralis* infection remains unclear, it has been suggested that IL-2 derived from CD4^+^ T helper cells may influence the function of ILC2s in lung inflammation [24, 29, 30]. Furthermore, a recent report also suggested that IL-9 played important roles in promoting ILC2 survival and function in helminth-induced lung inflammation [31].

The biological effect of IL-25 was mediated by the signaling pathways through a cognate receptor IL-17RB [32]. Several lines of evidence using IL-17RB deficient mice demonstrated the requirement of the IL-17RB receptor signaling in activating the expansion and function of ILC2 in response to allergens [18] and *N*. *brasiliensis* parasites [5]. In *N*. *brasiliensis* infection, ILC2s in IL-17RB deficient mice failed to expand during the early stage of infection but not in the late stage of infection. Indeed, we observed the reduction of ILC2s in the mesenteric lymph nodes of IL-17RB deficient mice at both early and late stage *T*. *spiralis* infection, implicating that the distinct pattern and function of IL-25-activated ILC2 may be induced differently in response to individual parasites that occupy different niches and possess unique life cycles. Furthermore, the period that antigen-specific Th2 and Th9 cytokine responses and their effector function to *T*. *spiralis* infection attenuated in IL-17RB deficient mice were correlated with the delayed worm expulsion in the intestines and muscle phase of infection. These data support our previous finding that the protective immune response against *T*. *spiralis* infection required IL-25-mediated Th2 and Th9 immune functions [17]. Interestingly, although IL-25 and IL-33 were both recognized as epithelial-derived cytokines promoting the expansion and function of ILC2s, they seem to have independent functions in responding to intestinal helminth parasites. In *Nippostrongylus brasiliensis* infection, ST2^−/−^ mice demonstrated normal Th2 cytokine response [33], whereas type-2 cytokine response generated after *T*. *spiralis* infection was shown to be dependent on ST2 [34]. Although intestinal worms were expelled normally in ST2^−/−^ mice, these mice exhibited the enhanced muscle larvae number, resulting from the compromised Th2 immune response [34]. It appears that intestinal helminth parasites may generally induce IL-25 in the intestinal epithelial cells that promote the cooperative function of innate ILC2 and adaptive T helper cells to augment antigen-specific Th2 and Th9 cytokine production, and that is essential for helminth parasite expulsion. Overall, these results are of particular significance as they suggest that IL-25 and IL-33 may play nonredundant roles in helminth infection and discrete pathways may be needed for host immune responses to different intestinal helminth parasite infections.

## Supporting information

S1 Fig The induction of Lin^-^IL-17RB^+^KLRG1^+^ST2^+^ cells in *T*. *spiralis*-infected mice. After *T*. *spiralis* infection for 7 days, the lamina propria cells of small intestine were subjected for the analysis of cell surface marker (ST2, KLRG1, and IL-17RB) in Lin^-^CD3^-^CD4^-^ cells by flow cytometry.
(TIF)

S2 Fig Frequency of CD3^+^CD4^+^ T cells in the small intestine of mice treated with anti-CD4 antibody. After *T*. *spiralis* infected mice were treated with rat IgG or anti-CD4 antibody every other day for 14 days, the lamina propria cells of small intestine were subjected for the analysis of CD3^+^CD4^+^ cells (A, B) and CD3^-^CD4^-^IL-17RB^+^CD127^+^T1/ST2^+^ (C, D) by flow cytometry and analyzed for the frequency and cell numbers. Data represent one of two independent experiments (n- = 3 mice per group).
(TIF)

## References

[1] Patel N, Kreider T, Urban JF Jr., Gause WC. Characterisation of effector mechanisms at the host:parasite interface during the immune response to tissue-dwelling intestinal nematode parasites. Int J Parasitol. 2009;39(1):13–21. Epub 2008/09/23. doi: 10.1016/j.ijpara.2008.08.003 ; PubMed Central PMCID: PMC2842902. 18804113 10.1016/j.ijpara.2008.08.003 PMC2842902

[2] Grencis RK, Humphreys NE, Bancroft AJ. Immunity to gastrointestinal nematodes: mechanisms and myths. Immunol Rev. 2014;260(1):183–205. Epub 2014/06/20. doi: 10.1111/imr.12188 ; PubMed Central PMCID: PMC4141702. 24942690 10.1111/imr.12188 PMC4141702

[3] Vallance BA, Croitoru K, Collins SM. T lymphocyte-dependent and -independent intestinal smooth muscle dysfunction in the T. spiralis-infected mouse. Am J Physiol. 1998;275(5 Pt 1):G1157–65. Epub 1998/11/14. . 9815046 10.1152/ajpgi.1998.275.5.G1157

[4] Vallance BA, Galeazzi F, Collins SM, Snider DP. CD4 T cells and major histocompatibility complex class II expression influence worm expulsion and increased intestinal muscle contraction during Trichinella spiralis infection. Infect Immun. 1999;67(11):6090–7. Epub 1999/10/26. ; PubMed Central PMCID: PMC96997. 10531271 10.1128/iai.67.11.6090-6097.1999 PMC96997

[5] Neill DR, Wong SH, Bellosi A, Flynn RJ, Daly M, Langford TK, et al Nuocytes represent a new innate effector leukocyte that mediates type-2 immunity. Nature. 2010;464(7293):1367–70. Epub 2010/03/05. doi: 10.1038/nature08900 ; PubMed Central PMCID: PMC2862165. 20200518 10.1038/nature08900 PMC2862165

[6] Price AE, Liang HE, Sullivan BM, Reinhardt RL, Eisley CJ, Erle DJ, et al Systemically dispersed innate IL-13-expressing cells in type 2 immunity. Proc Natl Acad Sci U S A. 2010;107(25):11489–94. Epub 2010/06/11. doi: 10.1073/pnas.1003988107 ; PubMed Central PMCID: PMC2895098. 20534524 10.1073/pnas.1003988107 PMC2895098

[7] Moro K, Yamada T, Tanabe M, Takeuchi T, Ikawa T, Kawamoto H, et al Innate production of T(H)2 cytokines by adipose tissue-associated c-Kit(+)Sca-1(+) lymphoid cells. Nature. 2010;463(7280):540–4. Epub 2009/12/22. doi: 10.1038/nature08636 . 20023630 10.1038/nature08636

[8] von Moltke J, Ji M, Liang HE, Locksley RM. Tuft-cell-derived IL-25 regulates an intestinal ILC2-epithelial response circuit. Nature. 2016;529(7585):221–5. Epub 2015/12/18. doi: 10.1038/nature16161 ; PubMed Central PMCID: PMC4830391. 26675736 10.1038/nature16161 PMC4830391

[9] Nussbaum JC, Van Dyken SJ, von Moltke J, Cheng LE, Mohapatra A, Molofsky AB, et al Type 2 innate lymphoid cells control eosinophil homeostasis. Nature. 2013;502(7470):245–8. Epub 2013/09/17. doi: 10.1038/nature12526 ; PubMed Central PMCID: PMC3795960. 24037376 10.1038/nature12526 PMC3795960

[10] Klose CS, Artis D. Innate lymphoid cells as regulators of immunity, inflammation and tissue homeostasis. Nat Immunol. 2016;17(7):765–74. Epub 2016/06/22. doi: 10.1038/ni.3489 . 27328006 10.1038/ni.3489

[11] Wong SH, Walker JA, Jolin HE, Drynan LF, Hams E, Camelo A, et al Transcription factor RORalpha is critical for nuocyte development. Nat Immunol. 2012;13(3):229–36. Epub 2012/01/24. doi: 10.1038/ni.2208 ; PubMed Central PMCID: PMC3343633. 22267218 10.1038/ni.2208 PMC3343633

[12] Oliphant CJ, Hwang YY, Walker JA, Salimi M, Wong SH, Brewer JM, et al MHCII-mediated dialog between group 2 innate lymphoid cells and CD4(+) T cells potentiates type 2 immunity and promotes parasitic helminth expulsion. Immunity. 2014;41(2):283–95. Epub 2014/08/05. doi: 10.1016/j.immuni.2014.06.016 ; PubMed Central PMCID: PMC4148706. 25088770 10.1016/j.immuni.2014.06.016 PMC4148706

[13] Gerbe F, Sidot E, Smyth DJ, Ohmoto M, Matsumoto I, Dardalhon V, et al Intestinal epithelial tuft cells initiate type 2 mucosal immunity to helminth parasites. Nature. 2016;529(7585):226–30. Epub 2016/01/15. doi: 10.1038/nature16527 . 26762460 10.1038/nature16527 PMC7614903

[14] Fallon PG, Ballantyne SJ, Mangan NE, Barlow JL, Dasvarma A, Hewett DR, et al Identification of an interleukin (IL)-25-dependent cell population that provides IL-4, IL-5, and IL-13 at the onset of helminth expulsion. J Exp Med. 2006;203(4):1105–16. Epub 2006/04/12. doi: 10.1084/jem.20051615 ; PubMed Central PMCID: PMC2118283. 16606668 10.1084/jem.20051615 PMC2118283

[15] Zhao A, Urban JF Jr., Sun R, Stiltz J, Morimoto M, Notari L, et al Critical role of IL-25 in nematode infection-induced alterations in intestinal function. J Immunol. 2010;185(11):6921–9. Epub 2010/10/27. doi: 10.4049/jimmunol.1000450 ; PubMed Central PMCID: PMC2988083. 20974983 10.4049/jimmunol.1000450 PMC2988083

[16] Owyang AM, Zaph C, Wilson EH, Guild KJ, McClanahan T, Miller HR, et al Interleukin 25 regulates type 2 cytokine-dependent immunity and limits chronic inflammation in the gastrointestinal tract. J Exp Med. 2006;203(4):843–9. Epub 2006/04/12. doi: 10.1084/jem.20051496 ; PubMed Central PMCID: PMC1800834. 16606667 10.1084/jem.20051496 PMC1800834

[17] Angkasekwinai P, Srimanote P, Wang YH, Pootong A, Sakolvaree Y, Pattanapanyasat K, et al Interleukin-25 (IL-25) promotes efficient protective immunity against *Trichinella spiralis* infection by enhancing the antigen-specific IL-9 response. Infect Immun. 2013;81(10):3731–41. Epub 2013/07/31. doi: 10.1128/IAI.00646-13 ; PubMed Central PMCID: PMC3811766. 23897610 10.1128/IAI.00646-13 PMC3811766

[18] Lee JB, Chen CY, Liu B, Mugge L, Angkasekwinai P, Facchinetti V, et al IL-25 and CD4(+) TH2 cells enhance type 2 innate lymphoid cell-derived IL-13 production, which promotes IgE-mediated experimental food allergy. J Allergy Clin Immunol. 2016;137(4):1216–25 e1-5. Epub 2015/11/13. doi: 10.1016/j.jaci.2015.09.019 ; PubMed Central PMCID: PMC4826796. 26560039 10.1016/j.jaci.2015.09.019 PMC4826796

[19] Pozio E, Khamboonruang C. Trichinellosis in Thailand: epidemiology and biochemical identification of the aethiological agent. Trop Med Parasitol. 1989;40(1):73–4. Epub 1989/03/01. . 2740731

[20] Scales HE, Ierna MX, Lawrence CE. The role of IL-4, IL-13 and IL-4Ralpha in the development of protective and pathological responses to *Trichinella spiralis*. Parasite Immunol. 2007;29(2):81–91. Epub 2007/01/24. doi: 10.1111/j.1365-3024.2006.00920.x . 17241396 10.1111/j.1365-3024.2006.00920.x

[21] Helmby H, Grencis RK. IL-18 regulates intestinal mastocytosis and Th2 cytokine production independently of IFN-gamma during *Trichinella spiralis* infection. J Immunol. 2002;169(5):2553–60. Epub 2002/08/24. . 12193725 10.4049/jimmunol.169.5.2553

[22] Angkasekwinai P, Park H, Wang YH, Chang SH, Corry DB, Liu YJ, et al Interleukin 25 promotes the initiation of proallergic type 2 responses. J Exp Med. 2007;204(7):1509–17. Epub 2007/06/15. doi: 10.1084/jem.20061675 ; PubMed Central PMCID: PMC2118650. 17562814 10.1084/jem.20061675 PMC2118650

[23] Chen CY, Lee JB, Liu B, Ohta S, Wang PY, Kartashov AV, et al Induction of Interleukin-9-Producing Mucosal Mast Cells Promotes Susceptibility to IgE-Mediated Experimental Food Allergy. Immunity. 2015;43(4):788–802. Epub 2015/09/28. doi: 10.1016/j.immuni.2015.08.020 ; PubMed Central PMCID: PMC4618257. 26410628 10.1016/j.immuni.2015.08.020 PMC4618257

[24] Liu B, Lee JB, Chen CY, Hershey GK, Wang YH. Collaborative interactions between type 2 innate lymphoid cells and antigen-specific CD4+ Th2 cells exacerbate murine allergic airway diseases with prominent eosinophilia. J Immunol. 2015;194(8):3583–93. Epub 2015/03/18. doi: 10.4049/jimmunol.1400951 ; PubMed Central PMCID: PMC4390517. 25780046 10.4049/jimmunol.1400951 PMC4390517

[25] Grencis RK, Riedlinger J, Wakelin D. L3T4-positive T lymphoblasts are responsible for transfer of immunity to *Trichinella spiralis* in mice. Immunology. 1985;56(2):213–8. Epub 1985/10/01. ; PubMed Central PMCID: PMC1453703. 3876979 PMC1453703

[26] Angkasekwinai P, Chang SH, Thapa M, Watarai H, Dong C. Regulation of IL-9 expression by IL-25 signaling. Nat Immunol. 2010;11(3):250–6. Epub 2010/02/16. doi: 10.1038/ni.1846 ; PubMed Central PMCID: PMC2827302. 20154671 10.1038/ni.1846 PMC2827302

[27] Wang YH, Angkasekwinai P, Lu N, Voo KS, Arima K, Hanabuchi S, et al IL-25 augments type 2 immune responses by enhancing the expansion and functions of TSLP-DC-activated Th2 memory cells. J Exp Med. 2007;204(8):1837–47. Epub 2007/07/20. doi: 10.1084/jem.20070406 ; PubMed Central PMCID: PMC2118667. 17635955 10.1084/jem.20070406 PMC2118667

[28] Huang Y, Guo L, Qiu J, Chen X, Hu-Li J, Siebenlist U, et al IL-25-responsive, lineage-negative KLRG1(hi) cells are multipotential 'inflammatory' type 2 innate lymphoid cells. Nat Immunol. 2015;16(2):161–9. Epub 2014/12/23. doi: 10.1038/ni.3078 ; PubMed Central PMCID: PMC4297567. 25531830 10.1038/ni.3078 PMC4297567

[29] Mirchandani AS, Besnard AG, Yip E, Scott C, Bain CC, Cerovic V, et al Type 2 innate lymphoid cells drive CD4+ Th2 cell responses. J Immunol. 2014;192(5):2442–8. Epub 2014/01/29. doi: 10.4049/jimmunol.1300974 . 24470502 10.4049/jimmunol.1300974

[30] Wilhelm C, Hirota K, Stieglitz B, Van Snick J, Tolaini M, Lahl K, et al An IL-9 fate reporter demonstrates the induction of an innate IL-9 response in lung inflammation. Nat Immunol. 2011;12(11):1071–7. Epub 2011/10/11. doi: 10.1038/ni.2133 ; PubMed Central PMCID: PMC3198843. 21983833 10.1038/ni.2133 PMC3198843

[31] Turner JE, Morrison PJ, Wilhelm C, Wilson M, Ahlfors H, Renauld JC, et al IL-9-mediated survival of type 2 innate lymphoid cells promotes damage control in helminth-induced lung inflammation. J Exp Med. 2013;210(13):2951–65. Epub 2013/11/20. doi: 10.1084/jem.20130071 ; PubMed Central PMCID: PMC3865473. 24249111 10.1084/jem.20130071 PMC3865473

[32] Rickel EA, Siegel LA, Yoon BR, Rottman JB, Kugler DG, Swart DA, et al Identification of functional roles for both IL-17RB and IL-17RA in mediating IL-25-induced activities. J Immunol. 2008;181(6):4299–310. Epub 2008/09/05. . 18768888 10.4049/jimmunol.181.6.4299

[33] Hoshino K, Kashiwamura S, Kuribayashi K, Kodama T, Tsujimura T, Nakanishi K, et al The absence of interleukin 1 receptor-related T1/ST2 does not affect T helper cell type 2 development and its effector function. J Exp Med. 1999;190(10):1541–8. Epub 1999/11/24. ; PubMed Central PMCID: PMC2195706. 10562328 10.1084/jem.190.10.1541 PMC2195706

[34] Scalfone LK, Nel HJ, Gagliardo LF, Cameron JL, Al-Shokri S, Leifer CA, et al Participation of MyD88 and interleukin-33 as innate drivers of Th2 immunity to *Trichinella spiralis*. Infect Immun. 2013;81(4):1354–63. Epub 2013/02/14. doi: 10.1128/IAI.01307-12 ; PubMed Central PMCID: PMC3639596. 23403558 10.1128/IAI.01307-12 PMC3639596

