# Supplementary figures and images for: ILC2s activated by IL-25 promote antigen-specific Th2 and Th9 functions that contribute to the control of Trichinella spiralis infection
Source: PLoS One. 2017 Sep 12;12(9):e0184684. doi: 10.1371/journal.pone.0184684 (PMC5595335; doi:10.1371/journal.pone.0184684)

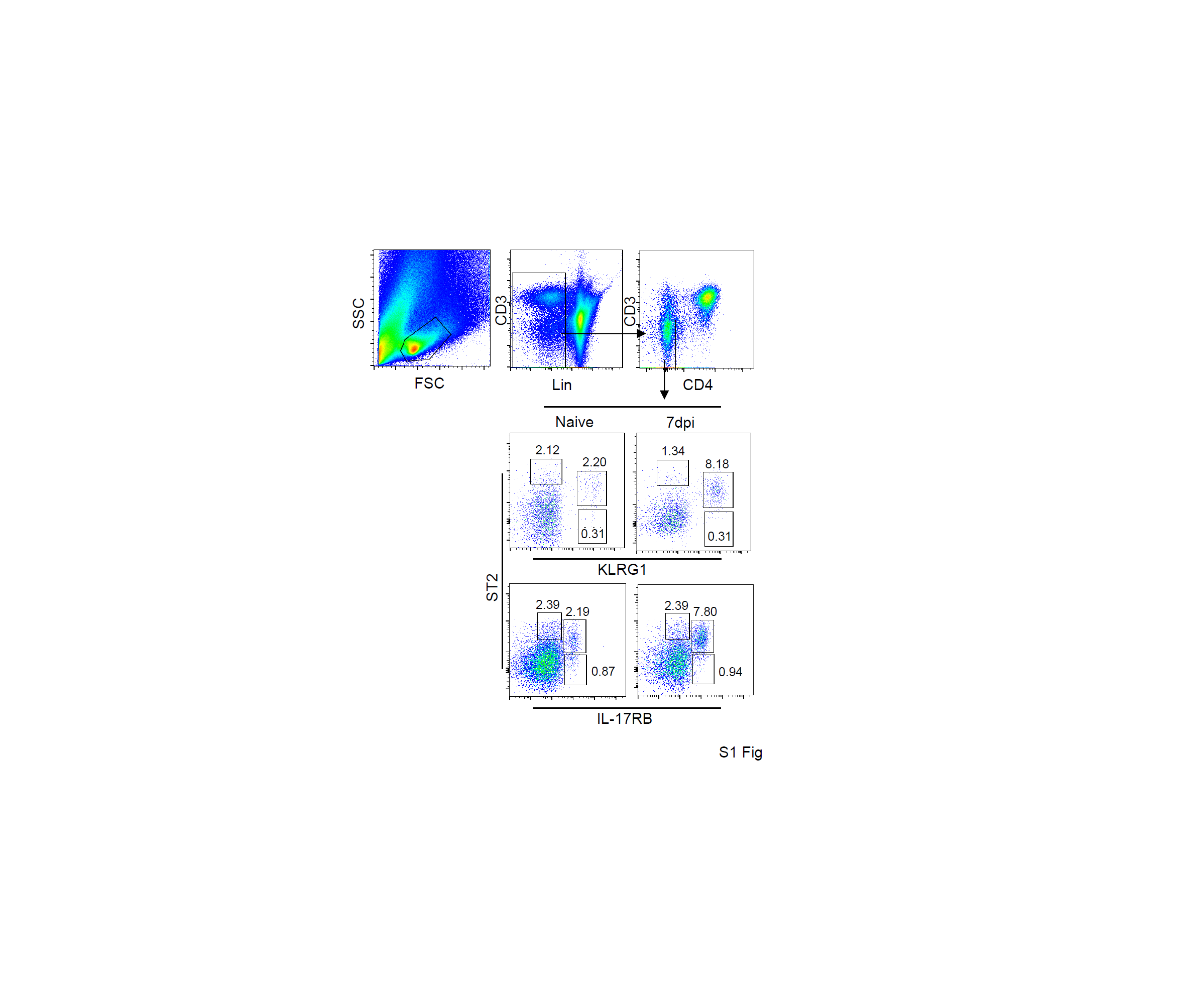

Supplement: S1 Fig — After T. spiralis infection for 7 days, the lamina propria cells of small intestine were subjected for the analysis of cell surface marker (ST2, KLRG1, and IL-17RB) in Lin-CD3-CD4- cells by flow cytometry. (TIF) [file pone.0184684.s001.tif]

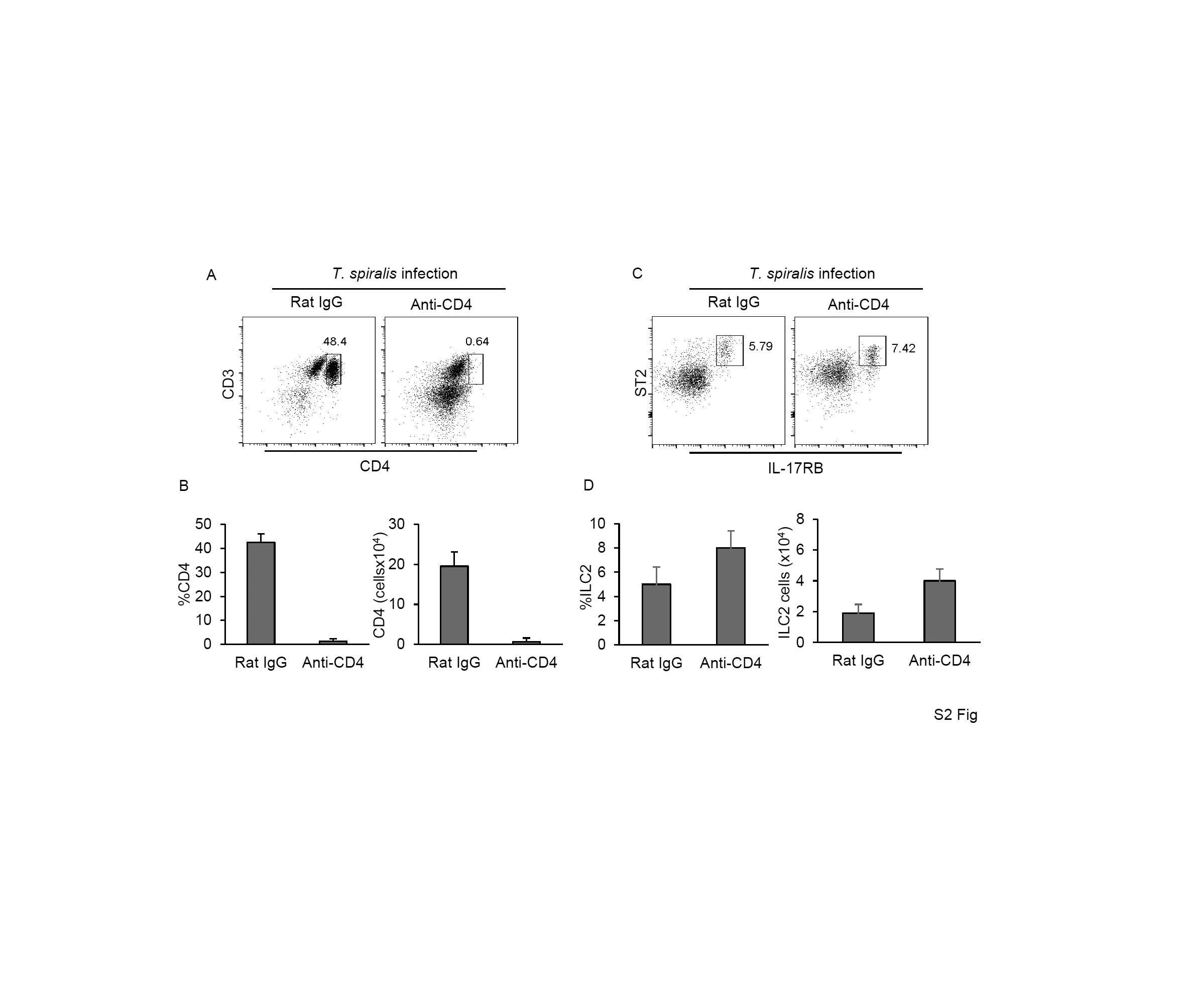

Supplement: S2 Fig — After T. spiralis infected mice were treated with rat IgG or anti-CD4 antibody every other day for 14 days, the lamina propria cells of small intestine were subjected for the analysis of CD3+CD4+ cells (A, B) and CD3-CD4-IL-17RB+CD127+T1/ST2+ (C, D) by flow cytometry and analyzed for the frequency and cell numbers. Data represent one of two independent experiments (n- = 3 mice per group). (TIF) [file pone.0184684.s002.tif]
